# Gene introduction approaches in chloroplast transformation and its applications

**DOI:** 10.1186/s43141-021-00255-7

**Published:** 2021-10-06

**Authors:** Asqwin Uthaya Kumar, Anna Pick Kiong Ling

**Affiliations:** Division of Applied Biomedical Sciences and Biotechnology, School of Health Sciences, International Medical University, 126 Jalan Jalil Perkasa 19, Bukit Jalil, 57000 Kuala Lumpur, Malaysia

**Keywords:** Biolistic, Chloroplast, PEG-mediated, Phytoremediation, Transformation, Vaccine

## Abstract

**Background:**

Chloroplast is a type of plastid that is believed to be originated from ancestral cyanobacteria. Chloroplast besides being a major component for photosynthesis, also takes part in another major plant metabolism, making it one of the major components of plants.

**Main body:**

Chloroplast transformation is an alternative and better genetic engineering approach compared to the nuclear transformation that has been widely applied in plant genetic engineering. Chloroplast transformation has exhibited various positive effects as compared to nuclear transformation. This is a more preferred technique by researchers. To carry out chloroplast transformation, the vector design must be performed, and a selectable marker needs to be incorporated before the chloroplast could uptake the construct. The common way of introducing a gene into the host, which is the chloroplast, involves the biolistic, PEG-mediated, carbon nanotubes carriers, UV-laser microbeam, and *Agrobacterium*-mediated transformation approaches. Apart from discussing the processes involved in introducing the gene into the chloroplast, this review also focuses on the various applications brought about by chloroplast transformation, particularly in the field of agriculture and environmental science.

**Conclusion:**

Chloroplast transformation has shown a lot of advantages and proven to be a better alternative compared to nuclear genome transformation. Further studies must be conducted to uncover new knowledge regarding chloroplast transformation as well as to discover its additional applications in the fields of biotechnology.

## Background

A plastid is a group of the diverged type of organelles that are commonly seen in any plant cells, algae, and a certain type of parasite species. It is presumed to be originated from cyanobacteria that have developed bacterial endosymbiont interaction with a eukaryotic host cell in the past [19–21]. Plastids are known for their ability to convert energy sources to metabolites and most known plastids are encompassed with genes that are coded for photosystem associating function [40]. Plastid, in general, is categorized by its behavior and complexity [24], and the commonly known plastid is archetypical plastid. Archetypical plastid is the organelle that contains photosynthetic cells such as chlorophyll. For instance, in the case of plants, the chloroplast is categorized as archetypical plastids [20, 24]. Chloroplast is one of the vital organelles found in plants that is differentiated from proplastids [14], and it plays a major role in photosynthesis where the light energy obtained directly from the sunlight is converted to biochemical energy, which is used to carry out the regular metabolic activities and growth of the plants [51, 56]. Other than the commonly known photosynthesis role of chloroplast, it also involves other fundamental plant cellular processes, such as amino acid production, nitrogen metabolism, secondary metabolites, and fatty acid production [51, 56]. The currently known chloroplast genome structures possess around 100 to 200 kb genes, which show comparatively the size of the chloroplast genome is smaller than its ancestor [14, 22].

Studies on chloroplast transformation have been conducted since its discovery 20 years ago, where it was identified as one of the effective substitutions to the conventional nuclear transformation [57, 59]. The chloroplast transformation method was identified as a perfect host for transgenes expression, in which most chloroplast genomes of angiosperm plants are obtained maternally, and eventually, the transgene dissemination is inhibited. This makes the chloroplast genome to be a powerful component in producing genetically modified plants [57, 59]. Compared to nuclear genome transformation, chloroplast transformation provides a larger yield in protein levels as evidenced in a study whereby around 46% of the protein that was not originated from within its species [57, 59] and proteins that are collected from chloroplast transformation are not infected with any virus or human pathogens [57]. This factor makes chloroplast transformation to be more attractive to the researchers especially in the discovery of protein productions.

## Main text

### Chloroplast transformation

Chloroplast transformation involves modification of the genome or introduction of a new foreign gene into the chloroplast. This process involves the designing of the DNA construct, including the selectable markers, as well as the introduction of the DNA construct to the chloroplast. Design of vector and selectable marker plays an important role in which the desired gene will be placed into the DNA construct according to the use of the chloroplast transformation and to identify the successfully transformed chloroplast. As for the introduction of genes, it involves the method of inserting the DNA construct into the chloroplast. These processes will be further discussed in the following sections.

### Designing vector and selectable markers

**Fig. 1:**
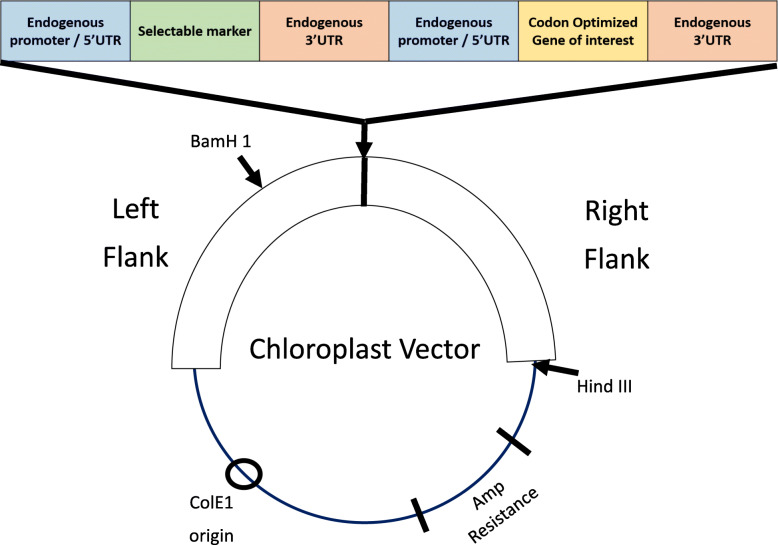
The design of a common chloroplast vector

Before the DNA construct or also known as a vector is introduced to the chloroplast, the DNA construct will need to undergo modification in its genomic sequence. Commonly, the gene of interest for the transformation and a selectable marker will be introduced to the genomic sequence of the vector. This gene of interest will be selected based on the functions of the plant after the transformation. Meanwhile, the main function of the selectable marker is to assist in the identification of transformants, whereby the selectable marker will be used to distinguish the transformants from the non-transformed chloroplast [11–13]. Figure 1 shows the common vector design used for most of the chloroplast transformation process.

In some plants’ chloroplast that is not capable of providing the sequence of regulation expression, 5′UTR and 3′UTR are obtained from other plants and the chloroplast is incorporated into the vector [11]. These cis-elements, which are the UTR, are obtained from endogenous plant genes that are able to express the genes in high amounts [13] or can be obtained from separate gene sources which are compatible [11, 13, 16]. There are two types of genome sequence designs—the single-gene expression refers to the vector design that is incorporated with one gene of interest while the multiple gene expression vector design involves the incorporation of multiple genes of interest with multiple protein-coding genes [1].

Selectable markers are the main identification system of transformants. There are various selectable markers with various selection mechanisms such as antibiotic resistance, herbicide resistance, metabolic markers, and photosynthetic genes [11–13, 16, 61]. One of the commonly used selectable markers in chloroplast transformation is the *aadA* cassette, which provides antibiotic resistance properties to the transformed chloroplast. This gene allows the transformant to be protected from the streptomycin antibacterial drug [13]. Another common selectable marker with an antibacterial resistance mechanism is the *aphA-6* gene, which enables the transformant to grow in the presence of any microbial that targets ribosomes [13]. Besides antibiotic resistance-based selectable marker, green fluorescent protein (*gfp*) is a common laboratory transformation identifying marker. This process includes the incorporation of the *gfp* gene into the vector, which allows the chloroplast callus to be transformed to exhibit fluorescent properties when placed in the dark. This is one of the easiest and cheapest genes that can be utilized as a selectable marker [11–13, 16]. Once the gene of interest and the selectable marker are incorporated into the DNA construct, the construct will proceed to the uptake of the chloroplast. The process is called the introduction of the gene to the host cell, which is the chloroplast.

### Introduction of gene

Once the vector for the chloroplast has been successfully constructed, it is introduced into the chloroplast for the transformation process. The transformation method indicates the introduction of desired new foreign genetic material into the host or targeted cells. In general, plant transformation can be carried out via direct or indirect gene transfer that works on different principles [35]. The direct gene transfer approach is known to involve physical or chemical reactions, while the indirect gene transfer method utilizes biological vectors to introduce the genes into the targeted cell/tissue [35]. The indirect method in chloroplast transformation is a novel technique, whereby alteration must be performed on the VirD2 protein of *Agrobacterium* that plays a major role in *Agrobacterium*-mediated transformation [39]. The following sections discuss the commonly used approaches in chloroplast transformation.

#### Biolistic method

The biolistic method is not something entirely new in the world of genetic engineering. It was first identified and introduced to the field in the late 1980s, where it was initially tested on plants for transformation studies [23, 38]. The word “biolistic” originated from the term biology, and ballistics refers to its mode of action that is similar to a gun [9, 23, 38]. The biolistic approach is one of the commonly used techniques in plant transformation, and other methods will only be considered when the biolistic approach is not suitable for the transformation process [38]. The whole concept of biolistic is depending on high pressure, whereby the desired DNA construct will be projected into the host using a gene gun at high speed with the help of pressurized gas [6, 9, 23, 38, 63].

The whole biolistic process begins with the purification of the gold/tungsten particle that is used as the delivery vector. The selected particle will undergo a purification process together with the treatment using isopropanol and glycerol to obtain the purest form of the particle [5]. This step ensures the metal particle used is free from contaminants or other foreign substances to prevent the DNA construct from being affected throughout the process. The DNA construct will be introduced to the metal particles [5, 6, 8, 9, 23, 63], where the DNA construct will coat the metal particle by using the precipitation principle [5] and the metal particles will be attracted to the DNA construct which is a negatively charged compound [9]. This step requires the use of calcium chloride and isopropyl for the formation of the DNA-tungsten / gold complex. The complex formed is isolated and placed on the macro-carrier known as a plastic bullet [5, 9]. The microcarrier is loaded into the chamber, where it is located around 7 inches away from the rupture disk [5, 9, 38]. After the macro-carrier is loaded into the biolistic chamber, the petri dish containing the desired chloroplast to be transformed is placed at the bottom of the biolistic chamber and the pressure within the chamber is reduced to the desired level [5, 8, 9, 38]. The pressure applied varies based on the type of cells used and the distance between the rupture disk and the Petri dish [5, 8, 29].

**Fig. 2:**
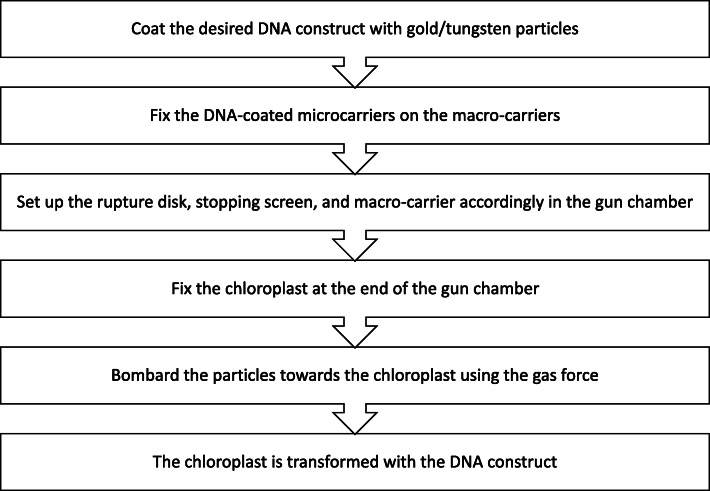
Flow chart that summarizes the steps involved in biolistic process

After this setup, the bombardment process can take place. Commonly, two methods will be used for the bombardment of the complex, one of which involves charged electricity and the other involves pressurized gas [9, 23, 38]. It is common to use helium gas, where pressurized helium gas is applied on the macro-carrier to transfer the complex into the chloroplast at high speed [5, 8, 9, 23, 29, 38, 39]. After the bombardment, the DNA construct is incorporated into the desired cells followed by 2 weeks of incubation for the healing of the cells to take place and further process is carried out once the callus formation is observed [5]. This whole biolistic process is summarized in Fig. 2.

The biolistic approach has been utilized on *Artemisia annua* to perform chloroplast genome transformation in one of the studies to produce a higher yield of artemisinin for a drug against malaria infection. This finding could lead to future studies on producing artemisinin on a larger yield for its global demand and this shows the success of the biolistic approach on chloroplast genome transformation in producing biopharmaceuticals [25]. In another study, the biolistic DNA delivery approach has efficiently worked on transforming chloroplast’s genome in potatoes. This study was performed to analyze the efficacy of transformation of macro-chloroplast, and the level of the heterologous protein produced is higher compared to the conventional approach [43].

The biolistic method was widely applied in plant transformation as it is not restricted to any certain species or plant cells [5, 6, 23]. It allows biolistic to be available for a wide range of transformation processes, and it is the most favorable approach for embryogenic callus [5]. Another factor that the biolistic method is frequently used is because of its capability in successfully transforming stable desired plastid gene, other than only focused on nuclear transformation [5]. Besides, in the biolistic method, the introduction of the desired genome to the targeted cells is performed by bypassing the cell wall protection and the gene transformed is not targeted or interrupted by any other cellular components on the cell surface, as the gene is directly introduced at the specific location of the desired cell [6, 9, 23].

Despite being the most widely used method, the biolistic approach has its drawbacks because the cost involved is relatively high as the equipment and chemicals used are expensive [23]. Besides, the chances of the targeted cell being damaged are high due to high pressurized penetration of the desired gene [6, 9, 23]. On top of that, due to the capability of transforming multiple genes at once within the targeted cell, the biolistic method might lead to gene silencing [5]. Despite all these advantages and disadvantages, the choice of utilizing the biolistic approach in gene introduction at the desired cell target is still very much dependent on the outcomes of the study.

#### Polyethylene glycol (PEG) method

Polyethylene glycol (PEG)-mediated transformation is the second most common plant transformation approach used widely in plant bioengineering. PEG is commonly used when the protoplast is the target cell for transformation, which makes it one of the common methods used in chloroplast transformation [35, 63]. It is one of the well-known plant or protoplast transformation techniques due to its straightforward utilization of equipment and minimal cost [32, 33, 37, 63]. The approach involves simple steps that can even be carried out in any laboratory setting, with the presence of a biosafety cabinet. Due to its effectiveness and ability to reproduce an expected outcome, this approach has become one of the preferred protoplast transformation techniques [32, 33, 37, 63]. PEG-mediated transformation introduces the gene of interest to the targeted cell through the disruption of the cell membrane’s dynamic by rising the permeability of the cell membrane [35]. In this process, the chloroplast will be co-cultured together with PEG, where the desired DNA construct will pass through the cell membrane in the form of vesicles to transform the chloroplast [63]. Recent studies reported that PEG has not shown convincing evidence in promoting the synergy between the DNA construct and the cell membrane; therefore, the function and process of PEG-mediated transformation remain unclear [33, 35]. Nevertheless, it has been hypothesized that PEG can control the osmotic condition of protoplast, which aids in the process of uptake of the DNA construct [35].

**Fig. 3:**
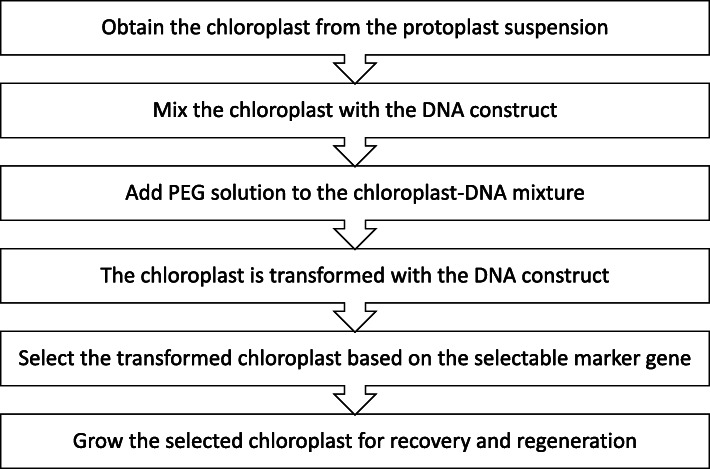
Flow chart that summarizes the steps involved in PEG-mediated transformation

The whole chloroplast transformation involves three important basic steps, i.e., (a) isolation of protoplast and chloroplast, (b) introduction of DNA construct into the chloroplast, and (c) regeneration of chloroplast [33]. The PEG-mediated transformation begins with the protoplast suspension acquisition by performing various enzymatic treatments upon the plant cells. The protoplast suspension is then allowed to undergo a centrifugation process [2, 32, 33, 36, 37, 41, 45, 48, 64]. Further purification step is taken to obtain chloroplast suspension which is free of contaminants and other cellular components. This step will ensure that the transformation is free from other plant cells and only chloroplast is transformed. The chloroplast pellet obtained from the centrifugation of chloroplast suspension after the purification step is then treated with MMg solution, which is a mixture of MES buffer, mannitol, and magnesium chloride [41]. After that, the chloroplast solution is mixed with the desired DNA construct that is incorporated with a selectable marker such as GFP. This was followed by the introduction of 60% PEG solution to the mixture and further incubated for a specific duration. The concentration of PEG solution and the incubation period is depending on the amount of the chloroplast and DNA construct used [2, 32, 33, 36, 37, 41, 45, 48, 64]. After 3 days of introducing PEG to the mixture, the chloroplast is grown in selectable conditions such as with the presence of antibiotics and antifungals, to avoid the growth of bacteria and fungi [64]. The transformants are monitored by the expression of the selectable marker incorporated in the DNA construct, and the transformation efficiency is also identified. Further confirmation on the identification of the transformants is performed by using PCR [2, 32, 33, 36, 37, 41, 45, 48, 64]. After the screening of the transformants, the transformed chloroplast is treated with osmotic buffer and sorbitol followed by the culturing of transformant on regeneration medium for recovery process [2, 32, 33, 36, 37, 41, 45, 48, 64]. This whole PEG-mediated transformation is summarized in Fig. 3.

The advantages of PEG-mediated transformation include the fact that a huge-sized DNA construct can be uptaken by the protoplast without causing any physical damages to the cell membrane or the protoplast. Studies have also shown that PEG-mediated transformation is able to produce a high transformation efficiency in protoplast [38]. Nevertheless, the main drawback of PEG-mediated transformation is the production of a huge amount of transient transformants due to the challenges and complicated processes that normally lead to retrieving a huge amount of active protoplast [2, 33].

#### Carbon nanotube carriers

Carbon nanotube carriers (CNT) are based on nanotechnology as discovered by Sumio Iijima in 1991 [3]. CNT are recognized as a potential delivery system for carrying the biomolecules and drug components to their targeted location [53]. The physical properties and their unique structure enable CNT to be a successful transporter [53]. The nanoparticle sizes of 10 to 20 nm with a positive charge are used to deliver the pDNA into the chloroplasts for higher efficiency as the charged nanoparticle has proven to be able to move across the chloroplast envelope, which is the barrier present at the entry of the chloroplast [42]. This uptake was hypothesized as the lipid exchange envelope penetration (LEEP), and the major contributor to this hypothesis is the charge of the nanoparticle [42]. However, it has also been reported the designed nanoparticles targeting the chloroplast with the aid of the peptide did not adhere to the LEEP mechanism. However, this is only observed in Arabidopsis leaf mesophyll cells with a more than 75% success rate [42]. It is important to note that CNT have to be designed with the ability to uptake the pDNA and subsequently deliver the pDNA to the chloroplast selectively. The process of delivery using CNT as reported by Kwak et al. [28] is quite straightforward, whereby the designed CNT was incubated together with *Arabidopsis thaliana* mesophyll protoplast to allow the uptake of pDNA. The transformed protoplast was then identified via the expression of the yellow fluorescence protein (YFP) in the chloroplast. This CNT delivery approach is cost-effective that does not require any complex specialized equipment [28].

#### UV-laser microbeam

UV-laser microbeam-mediated gene delivery is an enticing delivery system due to its large spatial control over the laser beam produced by the optical fiber [62]. There are four types of laser-assisted gene delivery, which are (a) optoinjection, (b) transfection by laser-induced stress waves, (c) photochemical internalization, and (d) selective cell targeting with light-absorbing particles. The UV laser beam that is directed to the cell leads to cell perforation and causes the formation of a hole on the cell membrane [62]. The hole, which is at the size of approximately 0.5 *μm* is a self-healing hole that will recover within 5 s. During this opening, the exogenous DNA or the pDNA is uptaken by the cells and allows the transformation process to take place [49]. Weber et al. [60] has introduced the DNA into the chloroplast using the UV laser microbeam approach in *Brassica napus* protoplast. The uptake using this approach was verified under the fluorescence microscope. However, this approach is not widely used due to the high cost involved in acquiring the equipment that is able to generate a laser beam with the dimensions of 100nm. On top of that, there are also chances of damage occurring to the cell and chloroplast due to the UV-laser radiation [49]. Up until now, there are not many chloroplast transformation studies that have been utilizing this approach. Thus, more studies are needed in understanding its efficacy.

#### *Agrobacterium*-mediated transformation

*Agrobacterium*-mediated transformation is based on one of the *Agrobacterium* species, *A. tumefaciens*. *A. tumefaciens* is capable of simulating the natural plant transformation process, making it one of the highly recommended non-invasive gene delivery systems. In this technique, the pathogen infects and subsequently transfers the gene of interest into the chloroplast of the host plant. This approach involves 5 steps where it begins with (a) signal recognition, (b) T-DNA processing, (c) T-DNA movement to host cell, (d) T-DNA integrating with the host genome, and (e) expression of T-DNA [46]. A. tumefaciens*A. tumefaciens* would attach to the plant cells and the gene products within the pathogen, which would be transferred to the T-DNA found in the tumor-inducing (Ti) plasmid. The T-DNA would integrate with the chloroplast genome via non-homologous recombination. After the integration of the gene of interest, the chloroplast further regenerates and proliferates [46]. Block et al. [7] have successfully transformed the chloramphenicol resistant genes into the chloroplast genome using the *Agrobacterium*-mediated transformation technique.

#### Applications of chloroplast transformation

As discussed earlier, chloroplast transformation offers a lot of benefits and potentials over the nuclear transformation in plant cells. With all the discoveries and advancements in chloroplast transformation, the approach can be used in various fields for the benefits of humankind and nature. Chloroplast transformation can be used for a variety of purposes, such as in the production of transgenic plants, particularly the abiotic and biotic stress tolerance plants, vaccines, biomaterials, and biopharmaceuticals as well as the phytoremediation process. The next section discusses the three most common applications of chloroplast transformation in the field of plant biotechnology.

#### Vaccine production

One of the huge achievements of modern medicine with the assistance of recombinant technology and immunology is the production of novel recombinant vaccines, which have huge improvements over the healthcare system to prohibit the occurrence of the diseases or eliminating some of them [10, 31]. One of the traditional approaches in the production of recombinant vaccines is using the bacterial cell lines or mammalian cell lines according to the type of the vaccines produced. However, these cells have drawbacks where the process requires a very high cost, and yet only a limited amount of transformants could be obtained ([1, 10, 30, 34, 58]b [31];). Besides, the chances of transmitting human pathogens are high when mammalian cells are used in the production of vaccines [58]. Practically, this is not feasible and uneconomical for recombinant vaccine production. Therefore, a substitute is required for the vector cells to produce recombinant vaccines with an economically stable approach and improved benefits.

Plant chloroplast is a very good option to overcome challenges faced by the traditional approach in recombinant vaccine production. Recombinant vaccines produced by plant chloroplast overcome the problems faced by the traditional approach where the cost of operation is relatively low. Compared to the traditional approach, the plants that have successfully grown with the transformed chloroplasts containing the vaccine components do not require any specific storage condition. It can be stored in room temperature and also can be transported conveniently to anywhere compared to the transformed mammalian or microbial cells [1, 10, 30, 58].

Furthermore, the post-translational modification can be performed in chloroplast transformation while it is not achievable in microbial cell lines. The plant chloroplasts are free from human pathogens that reduced the risk of contamination as plants are not the host of human pathogens [1, 10]. One of the successfully developed recombinant vaccines based on the chloroplast transformation is Cholera Toxin B Antigen, which was developed to target *Vibrio cholerae* [10, 30]. Antigens that are developed from the chloroplast transformation approach have exhibited the highest affinity to the *Vibrio cholerae* toxin receptors and could produce an immunological response [10]. Besides, the anthrax vaccine, plague vaccine, and tetanus vaccine were also successfully developed via the chloroplast transformation approach [10, 30, 34].

#### Abiotic and biotic stress tolerance

Plants do undergo extrinsic stress circumstances, which will affect the physiological development of the plants and might lead the plants to senescence [4, 17, 18, 47]. The stresses could be categorized into biotic and abiotic stress. Biotic stress indicates the stress was due to the plant’s vulnerability towards the biology components such as microbes and insects, while abiotic stress is indicating the exposure of the plants to a physically harsh environment such as climate change and exposure to chemicals like pesticides and herbicides [4, 17, 54]. These stresses need to be addressed as they affect the plants on a large scale as well as affecting the plant metabolic function and ultimately lead to the extinction of some plants due to their inability in tolerating these extrinsic stresses [4, 17, 18, 44, 47, 54].

Transgenic plants could be the alternative solution for this issue as the production of transgenic crops that can overcome the abiotic and biotic stress could result in higher and enhanced crop production [54]. With chloroplast transformation, there are some achievements that have been reported. For instance, eggplant and carrot have been modified to overcome high salinity [4]. In the study performed by Singh et al. [52], chloroplast transformation in eggplant has supported the idea of the transformation to overcome environmental abiotic and biotic stress. The findings also lead to a novel approach to pesticide resistance issues on chloroplast transformed plants. Singh et al. [52] applied the bombardment approach using the pPRV111A plastid vector carrying the *aadA* gene for the transformation studies. Kumar et al. [26] has performed plastid transformation in carrot-cultured cells by transforming the betaine aldehyde dehydrogenase (BADH) gene to produce a crop with tolerance to high salinity. This study is one of the first to announce the success of gene expression via chloroplast transformation in a crop that is not of tobacco origin. These studies not only indicate the possibility and the future of chloroplast transformation in enhancing the crop tolerance in abiotic and biotic stress, but they also indicate the success of the transformation in the expression of the gene of interest in the chloroplast genome to increase the tolerance.

#### Phytoremediation

Phytoremediation is the application of plants to convert the contaminants or remove them from the environment [1, 15, 27, 50]. In the current situation of developing industrial activities, soil and water pollution is a common issue where a huge amount of metal contaminant could be found in the environment [27, 50, 55]. The previous conventional approach in dealing with these metal pollutants in the environment cost a huge amount of expenses and can affect the microbial life and soil condition, and eventually the environment’s health [27]. To address this issue, phytoremediation is selected as the alternative approach as it involves low cost and the whole process is safe for the environment as well as the ecosystem [1]. However, there is a minor concern over this conventional approach, whereby plants are not capable of converting the toxic metal contaminants to a non-toxic component. Therefore, plants are required to undergo the modification in genomic level and chloroplast transformation is one of the approaches that would provide the ability of conversion of toxic metal contaminant to the plants [50].

Studies by Ruiz et al. [50] and Tangahu et al. [55] have shown that chelators are capable of uptaking metal contaminants, and they are frequently found in bacterial cells. Mercury is one of the common toxic metal contaminants that is converted by using the modified plants and the chelators used for the uptake of mercury is Metallothionein [50, 55]. Besides these chelators, two other enzymatic genes that are capable of converting toxic mercury contaminant to a non-toxic contaminant are mercuric ion reductase and organomercurial lyase. These three components are incorporated into the chloroplast genome and allowed to be expressed as an operon. The process has successfully been performed in modified tobacco plants, and mercury contaminants have been successfully converted as evidenced in some studies [1, 50, 55].

## Conclusion and future prospects

Chloroplast transformation has become a huge success since its discovery. Compared to nuclear genome transformation, chloroplast transformation has shown a lot of advantages where it was proven to be a better alternative offering more benefits. These advancements and discoveries in chloroplast transformation have shown that it can be used in various applications in the biotechnology field including agriculture, phytoremediation, and biopharmaceuticals. Up to date, the plants that have been successfully transformed are tobacco, carrot, lettuce, oilseed rape, and eggplant. Even with all these achievements, chloroplast transformation does have its drawbacks and limitations. Further studies must be conducted to uncover new knowledge regarding chloroplast transformation, as well as to discover its additional applications in the fields of biotechnology.

## Data Availability

Not applicable.

## Abbreviations

CTN: Carbon nanotube carriers
DNA: Deoxyribonucleic acid
*Gfp*: Green fluorescent protein
PEG: Polyethylene glycol
